# Understanding patient engagement in health system decision-making: a co-designed scoping review

**DOI:** 10.1186/s13643-019-0994-8

**Published:** 2019-04-18

**Authors:** Tamara L. McCarron, Karen Moffat, Gloria Wilkinson, Sandra Zelinsky, Jamie M. Boyd, Deborah White, Derek Hassay, Diane L. Lorenzetti, Nancy J. Marlett, Thomas Noseworthy

**Affiliations:** 1 The Department Community Health Sciences, Teaching Research and Wellness Building, 3280 Hospital Drive NW, Calgary, Alberta T2N 4N1 Canada; 2 O’Brien Institute for Public Health, Teaching Research and Wellness Building, 3280 Hospital Drive NW, Calgary, Alberta T2N 4N1 Canada; 3 Patient Co-investigators, Community Health Sciences, University of Calgary, Alberta, Canada; 4 Faculty of Nursing, University of Calgary in Qatar, PO Box 23133, Doha, Al Rayayan Al Forousiya Qatar; 5 Haskayne School of Business, 2500 University Dr. NW, Calgary, Alberta T2N 1N4 Canada; 6 Health Sciences Library, University of Calgary, 3330 Hospital Drive NW, Calgary, Alberta T2N 4N Canada

**Keywords:** Patients, Capacity building, Patient participation, Patient-centered, Decision-making

## Abstract

**Background:**

With healthcare striving to shift to a more person-centered delivery model, patient and family involvement must have a bigger role in shaping this. While many initiatives involving patients and family members focus on self-care, a broader understanding of patient participation is necessary. Ensuring a viable and sustainable critical number of qualified patients and family members to support this shift will be of utmost importance. The purpose of this study was to understand how health systems are intentionally investing in the training and skill development of patients and family members.

**Methods:**

Patient co-investigators and researchers conducted a scoping review of the existing literature on methods adopted by healthcare systems to build the skills and capacity of patients to participate in healthcare decision-making using a recognized methodological framework. Six electronic databases were searched to identify studies. Two independent reviewers screened titles and abstracts and full-text papers for inclusion. The research team independently extracted data. Any disagreements were resolved by achieving consensus through discussion. Quantitative and qualitative content synthesis, as well as a quality assessment, was conducted.

**Results:**

After eliminating duplicates, the search resulted in 9428 abstracts. Four hundred fifty-eight articles were reviewed and 15 articles were included. Four themes emerged: forums (33%), patient instructors (20%), workshops (33%), and co-design (13%). Four of the identified studies measured the impact and overall effectiveness of the respective programs. Examples of how patient and family members were supported (invested in) included advocacy training to support future involvement in engagement activities, a training program to conduct patient-led research, involvement in an immersive experience-based co-design initiative, and involvement in training pharmacy students. Overall, these studies found positive outcomes when patients and family members were recipients of these opportunities.

**Conclusions:**

The results of this scoping review demonstrate that an evidence base around programs to advance patient engagement is largely absent. An opportunity exists for further research to identify strategies and measures to support patient engagement in healthcare decision-making.

**Electronic supplementary material:**

The online version of this article (10.1186/s13643-019-0994-8) contains supplementary material, which is available to authorized users.

## Background

The concept of including patients in health and health system decision-making has been around for over 50 years [1]. Patient involvement in various aspects of healthcare, from improving healthcare quality to promoting patient safety, has emerged as a critical priority, but understanding how best to engage patients is not well understood [2–10]. This knowledge gap results in frustrating barriers for decision-makers looking to draw transferable lessons to inform the design of patient engagement programs and processes [11–13]. Coupled with challenges to the sustainability of healthcare and the need for innovative solutions, patient engagement has become central to improving both quality and delivery of services [14]. Research has demonstrated that patients who take part in their healthcare decisions are likely to also have better health outcomes [15]. Following this line of logic, we can assume patients who actively engage in opportunities to improve healthcare decision-making may have gaps in the education and the training required to participate as an equal partner. While many initiatives focus on patient self-care, a broader approach to patient participation is necessary to support the effective restructuring of healthcare delivery. This requires a critical number of qualified patients and family members who not only want to engage, but who are also qualified and confident to work in partnership with healthcare professionals and other stakeholders. This involves harnessing the skills and further building the capabilities of patients to support their participation in healthcare decision-making across the entire health system [14, 16–18]. This area of patient engagement is not well defined, and it is unclear what strategies are currently being implemented to promote the active engagement of patients in building their skills and capabilities. While there have been systematic reviews published that have explored patient engagement in research, these reviews have primarily focused on improving self-care [19] and improving shared decision-making [20]. Given this gap in understanding, we conducted a scoping review to systematically map out the research in this area. The objective of this study is to understand how health systems are intentionally investing in building the capacity and ability of patients to meaningfully participate in all aspects of healthcare decision-making. The following research question was formulated: How do health systems develop the ability and skills of patients and family members to engage in healthcare decision-making?

## Methods

### Protocol and research question

This scoping review protocol was developed using the methodological framework proposed by Arksey and O’Malley [21] and further enhanced by Levac et al. [22]. This review follows a six-stage methodological framework following these steps: (1) identify the research question, (2) identify relevant studies, (3) study selection, (4) charting the data, (5) collating, summarizing and reporting the results, and 6) stakeholder consultation [21, 22]. Despite some contention within the academic community as to whether the quality assessment should be conducted or not, quality assessment of included studies was completed [23, 24]. We used a modified SPICE (setting, population/perspective, intervention, comparison and evaluation) methodology to develop our research question [25]. Our protocol was drafted a priori using the Preferred Reporting Items for Systematic Reviews and Meta-analysis Protocols (PRISMA-P). The final protocol was posted on the Open Science Framework (https://osf.io/2ta74/). This protocol was uploaded on 7 September 2018 but is not registered or otherwise published. This review was completed in accordance with the scoping review reporting guidelines (PRISMA-ScR) [26].

### Co-design and patient co-investigators

This study utilized a co-design methodology, whereby members of the public, in this case, the patient and family community, were involved in the design of the research project from genesis to completion, including question development, data extraction, and interpretation [27, 28]. These patients were recruited based on their prior experiences participating in healthcare system decision-making and their ability to commit to each phase of this project. Three patient co-investigators and two researchers formed the project team. Co-investigators were given information 3 weeks prior to the meeting, highlighting the project goals and objectives and the high-level requirements needed to complete each step of the scoping review. At the initial meeting, the team discussed the research question and what was meant by the term “investment,” and determined that it was important to consider not only the traditional understanding of investing, which is primarily financial in nature (i.e., payment or expenses), but also the act of devoting time, effort, or energy to an endeavor.

### Information sources and search strategy

Search terms were debated with this enhanced understanding of investment in mind, and the project team created an exhaustive and wide-reaching list of search terms to adequately describe possible methods used by health systems to invest in patients. We designed the search strategy in collaboration with a librarian, to be broad and inclusive. The search strategy combined terms from three distinct themes: (1) investments (e.g., educating, learning, training), (2) influences (e.g., decision-making, self-efficacy, and innovation), and (3) areas of patient involvement (e.g., governance and co-design). The researcher and librarian systematically searched CINAHL, MEDLINE, EMBASE, Education Research Complete, Business Source Complete, and PsycINFO for studies published between January 1, 2000, and July 30, 2016. We limited the search strategy to studies written in English. The final search strategy for MEDLINE can be found in Additional file 1.

### Eligibility criteria

Studies were included if they (1) had an adult patient/consumer focus, (2) contained a description of an investment, (3) focused on programs/activities/events that were determined to have an impact on the participation of patients in healthcare, (4) showcased how patients/consumers engaged with other patients or the health system, and (5) incorporated investments that enable patients/consumers to participate in various healthcare roles. Studies were excluded if they (1) focused on investments to improve self-care; (2) did not involve or engage patients; (3) focused on children, animals, or family members; (4) did not report outcomes; or (5) were opinion pieces or letters to the editor.

### Study identification

All search results were merged into a reference management software program (EndNote X7).

The first 200 abstracts acted as a calibration process, enabling the project team to review and revise the inclusion criteria.

In order to best utilize the patient co-investigators, the first 1700 title and abstracts were reviewed (500 per patient co-investigator, duplicate review by the first author). The project team came together to review and discuss the results of the title/abstract review which provided further insights into the perspectives of the patient co-investigators and assisted the researcher and second reviewer with the approach taken to the remaining title and abstracts. Disagreements were resolved through consensus or by a third reviewer.

The first author provided the patient co-investigators with a training session on how to locate the articles flagged for review within the electronic databases. All articles were pulled for full-text review by the patient co-investigators.

The project team met again as a group to discuss and adjudicate the first 20 full-text articles selected for review. Any questions were answered, and the remainder of the steps was discussed. Selected articles were reviewed in duplicate by both patient investigators and the first author. As with the abstract review, any disagreements were resolved by a third reviewer.

### Quality assessment

The Mixed Methods Appraisal Tool (MMAT) developed by McGill University was used to assess the quality of the identified studies [29]. This tool was chosen for its ease of use, and ability to assess a diversity of study designs [30]. The tool is comprised of two parts (an initial screening section followed by a series of four questions) to simultaneously appraise and describe the methodological quality of mixed, qualitative, and quantitative study designs [29]. In part 2 of the tool, retained studies that are qualitative or quantitative in design can result in scores ranging from 25% (when no criterion is met) to 100% (having met all 4 criteria). Mixed methods studies can result in scores ranging from 25% (when no criterion is met) to 100% (having met three criteria). We assume a low-quality study as being one that only meets 25% (or 1 of 4 criteria), medium quality (meets 2 of 4 criteria), and high quality (meets 3 of 4 criteria). The quality of included studies was assessed in duplicate by two researchers. Studies were not excluded based on quality.

### Data extraction (charting)

Frequencies and percentages were calculated to describe the data. A predefined data extraction (charting) sheet was developed by the study team. The research team independently extracted data and discussed the results and continuously updated the data-charting form in an iterative process. Any disagreements were resolved by achieving consensus through discussion.

We extracted data on article characteristics (e.g., country of origin, author, outcomes), participant characteristics (e.g., type of participant and number), investment characteristics (type of investment and description), and patient motivations (if included), see Additional file 2.

#### Data synthesis

The research team sorted each of the studies into “investment” themes by using a modified constant comparative method developed by Glaser [31]. This method required that the research team placed each study into an investment theme; comparing each new study to the previous to determine if there was a new theme. This process was repeated until all studies were placed into a unique theme.

## Results

**Fig. 1:**
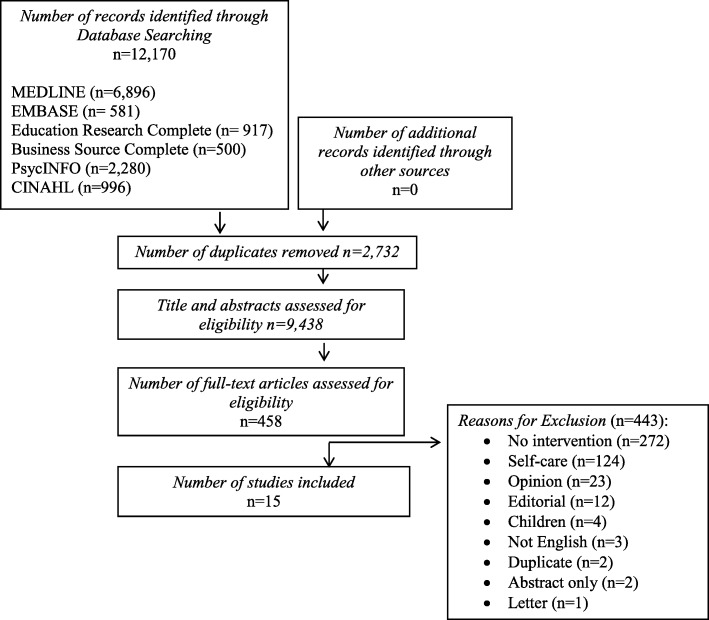
Study flow

The search resulted in 12,170 articles (Fig. 1). Duplicates (*n* = 2732) were removed and 9438 articles underwent title and abstract review. Four hundred fifty-eight articles were selected for full-text review. Of these, 15 studies were included in this scoping review, see Fig. 1.

### Study characterization

Of the 15 included studies, most (*n* = 8) were published after 2012 (Table 1). Studies were primarily published in the UK (*n* = 7) followed by the US (*n* = 3) with the fewest published in Canada, New Zealand, and Germany (*n* = 1). Nine of the studies utilized qualitative research methodologies to address their research questions, followed by mixed methods (*n* = 3) and quantitative non-randomized designs (*n* = 2).

**Table 1:** Study characteristics

Study characteristics (*n* = 15)	
	Count (%)
Year of publication	
2001–2005	4 (27)
2006–2011	3 (20)
2012–2016	8 (53)
Location of study	
UK	7 (7)
US	3 (20)
Australia	2 (13)
Canada	1 (7)
New Zealand	1 (7)
Germany	1 (7)
Study design	
Qualitative	9 (60)
Mixed methods	3 (20)
Quantitative (non-randomized)	2 (13)
Randomized control trial	1 (7)

### Quality assessment

Eight of the studies (7 qualitative and 1 quantitative) were deemed of high quality, and 3 studies (1 qualitative and 2 quantitative) were of medium quality. Factors that impacted the quality assessment were fairly consistent. The qualitative studies (*n* = 6) primarily did not discuss the role of the researcher (Q1.4), and two of the 2 quantitative research studies did not adequately report outcome data (Q2.3 and Q3.3). Four studies did not meet the initial screening questions and were not further assessed. Since we did not exclude based on quality studies not meeting the initial screening questions were not included in the presentation of outcomes (see Table 2).

**Table 2:**
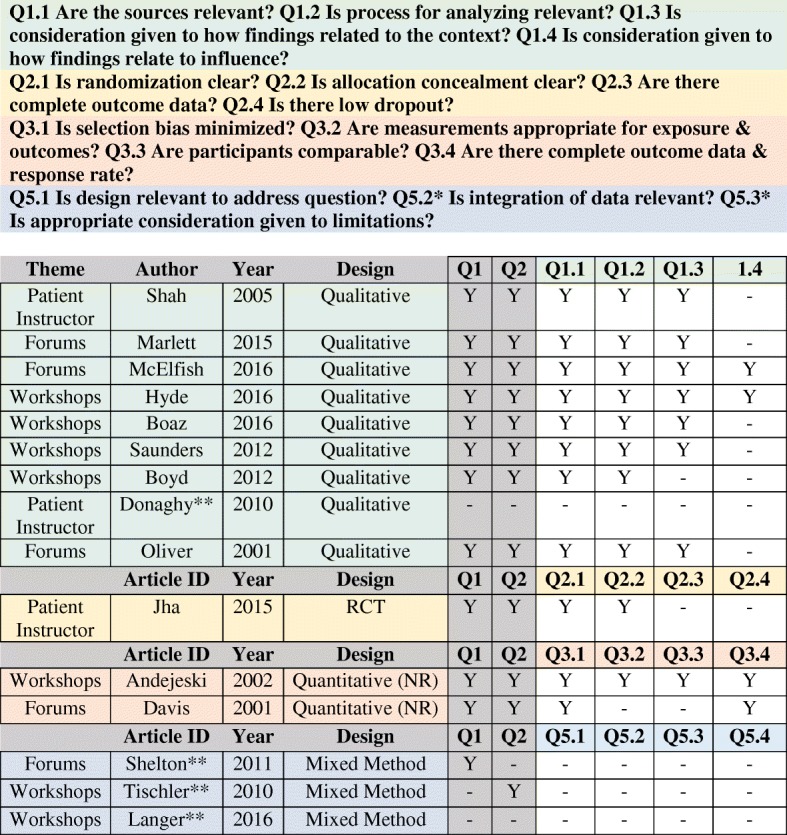
Mixed Methods Assessment Tool applied to included studies

*Both qualitative and quantitative components must be addressed

**Study not included in the presentation of outcomes

### Study themes

The included studies (*n* = 15) were themed into four categories by the research team including *forums*, *patient instructors*, *workshops*, and *co-design*. The first theme, *forums*, included five studies that highlight examples of participants receiving some form of instruction. These studies included examples of patients participating in a 1-year training program to be able to conduct patient engagement research [32], a series of informal training opportunities injected throughout a health technology assessment project [33], informal training offered to patients during stakeholder meetings to assist in developing a shared understanding of patient-centered outcomes [34], a 3-day advocacy training course to build confidence among breast cancer patients [35], and a 19-h 3-month training course for lay trainers to become patient navigators [36]. The second theme, *patient instructors*, expanded our definition of investment and included three studies that provided patient and family members with an opportunity to give an investment of time, as in providing patients with the opportunity and space to participate in healthcare delivery/health system improvements through their participation in student training [37–39]. The third theme, *workshops*, included five studies that highlight how patients are learning skills to participate in certain tasks. These studies included examples of opportunities for patients to participate in workshops to understand the systematic review process [40], a general information workshop to understand the research needs of cancer patients [41], an orientation presentation to enable patients to participate in a scientific review process [42], a series of workshops to help patients develop a common definition of patient-centeredness [43], and a series of learning opportunities to evaluate a collaborative learning model [44]. Finally, the fourth theme, *co-design*, included two studies involving patients who were involved in co-designing service delivery improvements: the first study involved patients in implementing improvement projects within acute hospital settings [45] and the second study co-designed improvements within a breast cancer service project [46]. See Table 3 for a complete description of the included studies.

**Table 3:** Summary of included studies

Theme	Author/year/location	Study design	Purpose of the study	Description of investment/no. of participants	Outcomes
Forums represent examples of individuals receiving some form of training	Marlett et al. [32] Canada	Qualitative	To describe a new role for patients who participate in a qualitative research training program	Patients participated in training so they could conduct patient experience research using qualitative methods. Twenty-one individuals participated in the forum	Training program increased the confidence and competence of patients to conduct research
	Oliver et al. [33] UK	Qualitative	To describe the methods used to involve patients in the Health Technology Assessment (HTA) process	Training opportunities were informally injected throughout the entire process. Including a 1-day “induction day” to kick off the project. No participant numbers were reported	None reported
	McElfish et al. [34] USA	Qualitative	To describe the process of developing patient-centered outcomes research with patients and community members	Informal training opportunities occurred throughout the project during the over 80 stakeholder meetings. Thirty-one community stakeholders participated in the forums	None reported
	Davis et al. [35] Australia	Quantitative non-randomized	To assess the effectiveness of an advocacy training program	A 3-day advocacy training course for breast cancer consumers. Fifty-one individuals participated in the forum	Patients receiving training had significantly increased involvement in advocacy activities
	^a^Shelton et al. [36] USA	Mixed methods	To compare the training-related experiences (knowledge, self-efficacy, satisfaction with training, and completion of role play-based training) of professional and lay trainers	An intensive training program provided over 19 h completed over the course of 3 months. Five individuals participated in the forum	None reported
Patient instructors are examples of patient and family members provide their own personal experiences in situations to improve medical training	Jha et al. [37] UK	RCT	To measure the impact of patient narratives as a method to train junior doctors in patient safety	Two learning sessions, collaboratively developed with patients. The sessions had a 15–18-min patient narrative and facilitated discussion between patients and trainees. Six patients and 5 carers participated	None reported
	Shah et al. [38] UK	Qualitative	To explore the patient experience of teaching undergraduate pharmacy students	Patients participated in education programs for pharmacy students. Thirty patients participated	Sharing experiences provided participants with a sense of worth and increased their overall confidence and self-esteem
	^a^Donaghty et al. [39] UK	Qualitative	To explore the perceptions of patient-led education for post-graduate trainees	Patients, with formal experience as teachers, designed a 1.5-h curriculum over a 1-month period. Three patients participated	None reported
Workshops are examples of how patients are learning skills to be able to participate in other tasks.	Hyde et al. [40] UK	Qualitative	To investigate the process and impact of involving patients in a systematic review	Patients participated in 3 information workshops on protocol design, interpreting results, and dissemination. Five patients participated	None reported
	Saunders et al. [41] Australia	Qualitative	To provide information on the research needs of cancer patients and to describe the priority setting process	Patients participated in a general information workshop. Thirty-two individuals participated	None reported
	Andejeski et al. [42] USA	Quantitative non-randomized	To evaluate the impact of having cancer survivors with advocacy experience participate as voting members of scientific review panels	Patient panel members received information and a presentation to orient them to the scientific review process. Eighty-five consumers participated	None reported
	^a^Tischler et al. [43] UK	Mixed methods	To establish a definition of patient-centeredness using abstracts from schizophrenia research and to explore the experiences of both psychiatrists and service users taking part in the research	Patients participated in 3 half-day workshops to define patient-centered care. Thirteen individuals participated	None reported
	^a^Langer et al. [44] UK	Mixed methods	To evaluate the patient TIPS collaborative learning model to patient and family and clinicians	Two exploration style focus groups, 3 orientation sessions, and 3 workshops (4 h) focusing on medical error. Nine family members completed the workshops	None reported
Co-designs are examples of involving patients in co-designing program improvements	Boaz et al. [45] UK	Qualitative	To explore the different roles adopted by patients after participation in quality improvement interventions	Small co-designed groups work on implementing improvements over 3–4 months	Three of 63 patients continued their involvement after project completion
	Boyd et al. [46] New Zealand	Qualitative	To describe how co-design can be used to improve patient experience with healthcare services	Embedded throughout the entire co-design process including surveys and workshops	None reported

^a^Study not included in the presentation of outcomes

### Study outcomes

Four of the 15 included studies explored the impact of the respective investments on increasing patient engagement in healthcare decision-making. Two studies discussed the impact of forums on patient involvement. The first study found that patients who had received this investment of training had significantly increased their involvement in advocacy activities, such as acting as a community board member [35]. The second study found that a 1-year training program increased the confidence and competence of patients to conduct health research [32]. The third study discussed the impact of patient instructors and found that the opportunity to share the patient experience with pharmacy students provided participants with a sense of worth and increased their overall confidence and self-esteem [38]. Lastly, the fourth study discussed the impact of co-design on patient involvement finding three of 63 patients continued their involvement after the project was complete [45]. Overall, these studies found positive outcomes resulting from one of these four investments.

## Discussion

Acknowledging the complexity of patient engagement, we undertook this scoping review to explore the nature and impact of investments implemented by health systems to build the capacity and ability of individuals to meaningfully participate in healthcare decision-making. We identified 15 diverse studies and four investment themes: (1) *forums*, (2) *patient instructors*, (3) *workshops*, and (4) *co-design*. Four of the 15 included studies evaluated the impact of programs designed to increase patient engagement in healthcare. The results of this scoping review indicate there is an opportunity for future research to further establish and evaluate programs that facilitate patient involvement. During this review, the authors noted that there were a number of gaps in the literature such as training to build additional competencies, such as governance experience; removing participation barriers for patients, such as providing financial assistance for expenses, including child care; and creating roles or opportunities for patients to develop new skills or further develop their existing skills.

This review is the first to comprehensively assess how health systems are investing in building the capacity and ability of patients. A number of benefits have been reported in previous studies that primarily focus on promoting self-efficacy and empowering and equipping patients with the skills and confidence to manage their own self-care [47–49]. Although enabling patient self-care is essential for the delivery of efficient and effective healthcare, opportunities exist to expand into other areas of patient engagement. The types of investments suggest there may be value in shifting the focus from patient engagement in self-care to an exploration of other ways in which health professionals and healthcare systems can benefit from engaging patients in healthcare governance and the establishment of system-level priorities.

While significant funding has been allocated to advance the inclusion of patients in healthcare decision-making, there is a lack of quality evidence to assess the transferability of various approaches to patient engagement in other settings. This lack of rigorous research may be contributing to the general absence of system-wide adoption of initiatives to encourage broader patient engagement in healthcare decision-making. There is a need to invest in interventions that evaluate the impact and effectiveness of these programs. Public-private partnerships such as the European Patients’ Academy (EUPATI) provide training opportunities for patients to increase their capacity and capability to contribute to health research [50]. Although the mission of the EUPATI initiative is that these interventions will translate into a new paradigm of increased patient involvement across the entire health research spectrum, it is still early to tell [51]. The Health Technology Assessment International (HTAi) [52] and James Lind Alliance [53] have resources available for patients and the public but do not provide tailored training opportunities. Studies further exploring the impact of these efforts should be undertaken.

The manner in which we approached this review served a dual purpose of both assessing the extent of the literature on patient engagement and providing a real-world opportunity to develop the capacity of patients to participate in this type of research. The patient co-investigators experienced an increased level of confidence in their abilities to participate in a scoping review as a result of this experience. In addition, all three patient co-investigators have sought additional opportunities for their involvement.

### Strengths and limitations

This study has strengths and limitations. We limited our search to English language peer-reviewed publications. As a result, it is possible that a search that sought to identify gray literature and research published in languages other than English may have yielded additional studies of relevance to this review. Due to the comprehensive search strategy, the volume of studies required us to limit publication dates of the included studies. While people have been thinking about engagement for over 50 years, it is important for programs that envision engagement beyond self-care be realized [2, 54]. This scoping review considered only investments from the patient perspective but it is also important to understand how health professionals are being supported to enable and support opportunities for patients. The inclusion of literature that focused on engagement strategies aimed at health professionals could have identified additional approaches to involving stakeholders in healthcare decision-making. Lastly, while the inclusion of patient co-investigators in the evidence synthesis could be perceived as design bias, we feel that the robust methodological processes we developed to conduct this review minimized any potential for bias, while supporting greater understanding and confidence among the patient co-investigators.

## Conclusion

While significant research exists that highlights how health systems are working with patients to better manage their own care, studies that explore other dimensions of patient engagement are largely absent. This study identified a few examples of how health systems are investing in building the capacity of patients. Creating opportunities for training and skill building, in all aspects of healthcare, enables patients to see first-hand the challenges faced by the healthcare system. This perspective helps to establish the role of the patient as a valued partner in healthcare decision-making. The results of this review suggest that achieving person-centered care may still be a long time away.

## Additional files

Additional file 1: Final MEDLINE Search Strategy. (DOCX 15 kb)

Additional file 2: Data-charting (extraction) sheet. (XLSX 9 kb)

## Abbreviations

PRISMA-P: Preferred Reporting Items for Systematic review and Meta-Analysis Protocols
PRISMA-ScR: PRISMA extension for scoping reviews
SPICE: Setting, population/perspective, intervention, comparison and evaluation
MMAT: Mixed Methods Appraisal Tool
