# Supplementary material for: Understanding patient engagement in health system decision-making: a co-designed scoping review
Source: Syst Rev. 2019 Apr 18;8:97. doi: 10.1186/s13643-019-0994-8 (PMC6474065; doi:10.1186/s13643-019-0994-8)
Supplement: Supplementary file 1 — Final MEDLINE Search Strategy. (DOCX 15 kb) [file 13643_2019_994_MOESM1_ESM.docx]

Appendix Final MEDLINE Search Strategy

(Literature search performed on July 30, 2016)

1. ((patient* or consumer*) adj5 (leadership skills or educating or education or learning or training or (research adj5 (involv* or engag*)) or professional development or capacity building or reimbursement* or incentive* or honorarium* or compensation or conference attendance or stipend*)).tw,kw.
2. (advocacy or decision making or self-efficacy or innovation or quality improvement or cost-effective* or cost efficien* or literacy or social change or cost benefit* or self-care or self-management or quality improvement* or personali* care or person cent* care or consumer cent* care or individual care or coping skills or patient experience or patient led research or user led research or sense of coherence or collaborative research or patient generated data or evidence-based change or patient satisfaction or patient safety or consumer safety).tw,kw.
3. 1 and 2
4. (expert patient* or patient activation).tw,kw.
5. ((patient* or consumer*) adj5 (governance or governing board* or co-design)).tw,kw.
6. 3 or 4 or 5
7. limit 6 to yr="2000 -Current"
8. limit 7 to (editorial or letter)
9. 7 not 8
10. limit 9 to animals
11. limit 9 to (animals and humans)
12. 10 not 11
13. 9 not 12
14. Limit 13 to English language
15. Limit 14 to “Review articles”
16. 16 or 17
17. Limit 14 to systematic reviews
18. 16 or 17
19. Limit 16 to randomized controlled trial
20. (randomized controlled trial or rct).tw.
21. 16 and 20
22. 19 or 21
23. Limit 16 to clinical trial, all
24. Limit 16 to observational study
25. Exp qualitative research
26. 18 and 26
27. (qualitative or interview* or focus group* or grounded theory or phenomenolog*).tw,kw.
28. 16 and 28
29. 27 or 29
30. (survey or surveys).tw,kw
31. 16 and 31
32. (cohort or case control).tw, kw
33. 18 and 33
